# Factors associated with relapse/progression in pediatric trunk and extremity rhabdomyosarcoma

**DOI:** 10.3389/fonc.2026.1866742

**Published:** 2026-07-02

**Authors:** Yanhua Li, Huanhuan Zhang, Jingbo Shao, Xuelian Liao, Yangyang Jiao, Shayi Jiang, Jingwei Yang

**Affiliations:** 1 Department of Hematology and Oncology, Shanghai Children’s Hospital, Shanghai Jiao Tong University School of Medicine, Shanghai, China; 2 Department of Radiology, Shanghai Children’s Hospital, Shanghai Jiao Tong University School of Medicine, Shanghai, China

**Keywords:** extremity, pediatric, progression, relapse, rhabdomyosarcoma, trunk

## Abstract

**Background:**

Rhabdomyosarcoma (RMS) of the trunk and extremities is associated with unfavorable outcomes due to high rates of metastasis and relapse. However, large-scale studies focusing specifically on this anatomical subsite remain limited. This study aimed to investigate the clinical characteristics and risk factors associated with relapse/progression in pediatric patients with trunk and extremity RMS.

**Purpose:**

To investigate the clinical features and risk factors associated with relapse/progression in pediatric patients with rhabdomyosarcoma (RMS) of the trunk and extremities.

**Methods:**

A retrospective analysis was conducted on clinical data from 15 children with trunk and extremity RMS treated at Shanghai Children’s Hospital between January 2011 and December 2024. All patients received multimodal therapy, including surgery, chemotherapy, and radiotherapy. Associations between clinical characteristics and relapse/progression rates were analyzed using descriptive statistics and Fisher’s exact test.

**Results:**

The median follow-up duration was 48 months. The 5-year event-free survival (EFS) was 60% (95% CI: 34.5%-85.5%), and the 5-year overall survival (OS) was 66.7% (95% CI: 42.1%-91.3%). The overall relapse/progression rate was 40% (95% CI: 16.8%-68.7%). Significantly higher relapse/progression rates were observed in patients with metastasis at diagnosis (85.7% vs 0%, *P<0.001*), high-risk stratification (75.0% vs 0%, *P=0.010*), and macroscopic residual disease after surgery (100% vs 10.0%, *P<0.001*). Regional lymph node involvement showed a trend toward a higher relapse/progression rate (66.7%) compared to no involvement (22.2%), although the difference was not statistically significant (*P=0.123*).

**Conclusion:**

Pediatric trunk and extremity RMS is associated with a high risk of relapse/progression. Metastasis at diagnosis and macroscopic residual tumor after resection are major adverse prognostic factors. Regional lymph node involvement may confer an increased risk, warranting validation in larger cohorts.

## 1 Introduction

Rhabdomyosarcoma (RMS) is the most common soft-tissue sarcoma in children, accounting for approximately two-thirds of pediatric sarcomas and 7%-8% of all malignant solid tumors in this population (1, 2). In the past decade, the fine-tuning of multimodal chemotherapy regimens has greatly improved the relapse free survival for patients with localized diseases. Currently, 70% of the patients survive with no relapse (3). Trunk and extremity rhabdomyosarcoma accounts for 15%-20% of RMS cases, occurs more frequently in older children, and is associated with relatively unfavorable outcomes due to higher rates of regional lymph node involvement, metastasis, and a higher proportion of alveolar histology (4). Due to its rarity, large-scale single-center studies on trunk and extremity RMS remain limited. This retrospective study aimed to analyze clinical data from 15 pediatric patients to identify factors associated with relapse/progression in childhood trunk and extremity RMS.

## 2 Materials and methods

### 2.1 Study population

Fifteen pediatric patients with trunk or extremity RMS treated at Shanghai Children’s Hospital between January 2011 and December 2024 were enrolled. All patients were under 18 years of age, with a definitive histopathological diagnosis of RMS confirmed by two independent pathology centers. For patients diagnosed with alveolar RMS after January 2019, *FOXO1* translocation status was evaluated by fluorescence *in situ* hybridization. The study protocol was approved by the Ethics Committee of Shanghai Children’s Hospital (No. 2025R201-E01).

### 2.2 Diagnosis and risk stratification

Pathological diagnosis and subtyping were performed according to the 2020 World Health Organization Classification of Soft Tissue and Bone Tumors (5). Patients enrolled prior to 2019 received treatment according to the Rs-99 protocol, with risk stratification based on the IRS grouping Classification (6). For patients enrolled after January 2019, the Rs-2018 protocol was used. Risk stratification, which was based on UICC-TNM staging, IRS grouping, and histology, was slightly modified from the criteria of the Children’s Oncology Group (COG) Soft Tissue Sarcoma (STS) Committee’s sixth clinical study (COG-STS-VI) (7, 8). Specifically, embryonal RMS (ERMS) patients with the following stage and group (Stage 1, Group III and Stage 3, Group I/II) were reassigned to the intermediate-risk (IR) group. The ongoing RMS clinical trial ARST1431(NCT02567435) conducted by the COG has also categorized these two subsets of patients as IR-RMS (9).

### 2.3 Treatment

All patients received multimodal therapy, including surgery, chemotherapy, and radiotherapy. The surgical goal was complete tumor resection whenever feasible. For unresectable tumors or cases requiring limb salvage, biopsy followed by neoadjuvant chemotherapy and delayed primary excision (DPE) was performed (10). Radiotherapy was administered to all patients, with doses determined by *FOXO1* fusion status, lymph node involvement, and IRS group (9). All patients received chemotherapy according to their risk group, to either the Rs-99 or the Rs-2018 protocol. The Rs-99 regimen involved alternating cycles of DVCP and IVE. Patients in the Low-risk (LR), IR, and high-risk (HR) groups received 6, 12 and 18 cycles of chemotherapy, respectively (6). The Rs-2018 regimen was administered as follows: the LR group received 9 cycles of the VAC/VA regimen; the IR group received alternating DVAC/IVE followed by VAC/AEV; the HR group received the IR regimen with the addition of irinotecan and sirolimus (11) (**Appendix A**).

### 2.4 Response evaluation and follow-up

Treatment response was evaluated according to RECIST 1.1 criteria: complete response (CR), partial response (PR), progressive disease (PD), and relapse (12). Follow-up evaluations were performed every 3 months for the first 2 years, every 6 months for the third year, and annually thereafter. Event-free survival (EFS) was defined as the time from diagnosis to disease progression, relapse, or death. Overall survival (OS) was defined as the time from diagnosis to death from any cause. The cutoff date for follow-up was June 30, 2025.

### 2.5 Analysis of clinical characteristics and relapse/progression rates

We analyzed the associations between clinicopathological factors and relapse/progression rates, including sex, histological subtype, local invasion of the primary tumor, maximum diameter of the primary tumor, regional lymph node involvement, metastasis, and treatment status. For patients in the high-risk group, the Oberlin score was calculated based on the following factors: age >10 years or <1 years, unfavorable primary tumor site, ≥3 metastatic sites, and bone or bone marrow involvement (13).

### 2.6 Statistical analysis

Statistical analysis was performed using SPSS 27.0 (released 2020, IBM Corp, Armonk, NY, USA). Categorical variables were presented as counts and percentages (n, %). The entire cohort survival curves were constructed using the Kaplan-Meier method. Between-group comparisons were performed using Fisher’s exact test. A two-sided *P<0.05* was considered statistically significant.

## 3 Results

### 3.1 Clinical characteristics

Fifteen patients were enrolled, including 8 males and 7 females. The median age at diagnosis was 48 months (range: 10–108 months). Primary sites included the trunk (4/15, 26.7%), upper extremity (2/15, 13.3%), and lower extremity (9/15, 60%). Histological subtypes were embryonal RMS (9/15, 60%) and alveolar RMS (6/15, 40%). Among alveolar cases, 3 were *FOXO1* positive. Tumor size >5 cm was present in 9 patients (60%). Regional lymph node involvement was detected in 6 patients (40%). metastasis was present in 7 patients (46.7%), most commonly to bone (6/7). According to TNM staging, 2 patients were stage II, 6 were stage III, and 7 were stage IV. By IRS grouping, 5 were group II, 3 were group III, and 7 were group IV. Seven patients (46.7%) were stratified as intermediate-risk, and 8 (53.3%) as high-risk. Among high-risk patients, 2 had an Oberlin score ≤1, and 6 had a score ≥2. Clinical data for the 15 patients are presented in **Table 1**.

**Table 1:** Clinical characteristics of 15 patients with trunk and extremity rhabdomyosarcoma.

Case No./sex/age (m)	Primary site	Histology	N	M	Group	Risk-group	Surgery time	Surgery	Outcome	F/U (m)
1/m/36	Trunk	ERMS	N1	M1	IV	HR	DPE	R2	DOD	14
2/f/48	Lower limb	ERMS	N0	M0	II	IR	upfront resection	R1	CDF	102
3/f/108	Trunk	ERMS	N1	M1	IV	HR	DPE	R2	DOD	15
4/f/15	Lower limb	ERMS	N0	M0	III	IR	DPE	R0	CDF	71
5/f/31	Lower limb	ERMS	N0	M0	II	IR	upfront resection	R1	CDF	48
6/f/48	Trunk	ERMS	N0	M0	III	IR	DPE	R1	CDF	88
7/m/14	Upper limb	ERMS	N0	M1	IV	HR	upfront resection	R0	DOD	27
8/f/96	Lower limb	ERMS	N0	M0	II	IR	upfront resection	R1	CDF	79
9/m/30	Lower limb	ERMS	N0	M1	IV	HR	DPE	R2	CDF	81
10/m/33	Trunk	ARMS	N1	M0	II	IR	upfront resection	R0	CDF	81
11/m/10	Lower limb	ARMS	N0	M0	II	IR	upfront resection	R1	CDF	175
12/f/96	Upper limb	ARMS	N1	M1	IV	HR	biopsy only	R2	DOD	30
13/f/85	Lower limb	ARMS	N0	M1	IV	HR	DPE	R1	CDF	21
14/m/49	Lower limb	ARMS	N1	M0	III	HR	DPE	R1	CDF	34
15/m/68	Lower limb	ARMS	N1	M1	IV	HR	biopsy only	R2	DOD	24

ERMS, embryonal RMS; ARMS, alveolar RMS; N1, Imaging or pathology shows regional lymph node involvement; M1, metastasis; HR, high-risk group; IR, Intermediate-risk group; DPE, delayed primary tumor excision; R0, microscopically negative surgical margins; R1, microscopically positive surgical margins; R2, surgically macroscopic residual disease; CDF, continuous disease free; DOD, died of the disease.

### 3.2 Treatment

All patients completed treatment and follow-up. Primary tumor resection included upfront resection in 6 patients (40%), DPE in 7 (46.7%), and biopsy only in 2 (13.3%). Among the 8 patients with localized disease, 2 achieved R0 (microscopically negative surgical margins) resection and 6 achieved R1 (microscopically positive surgical margins) resection. Among 7 patients with metastatic disease, 1 achieved R0, 1 achieved R1, and 5 achieved R2 (surgically macroscopic residual disease) resection. All patients received systemic chemotherapy. Of these, 9 (60%) were treated with the Rs-2018 protocol and 6 (40%) with the Rs-99 protocol. Radiotherapy was administered to all patients at the primary and metastatic sites.

### 3.3 Relapse/progression analysis

**Figure 1:**
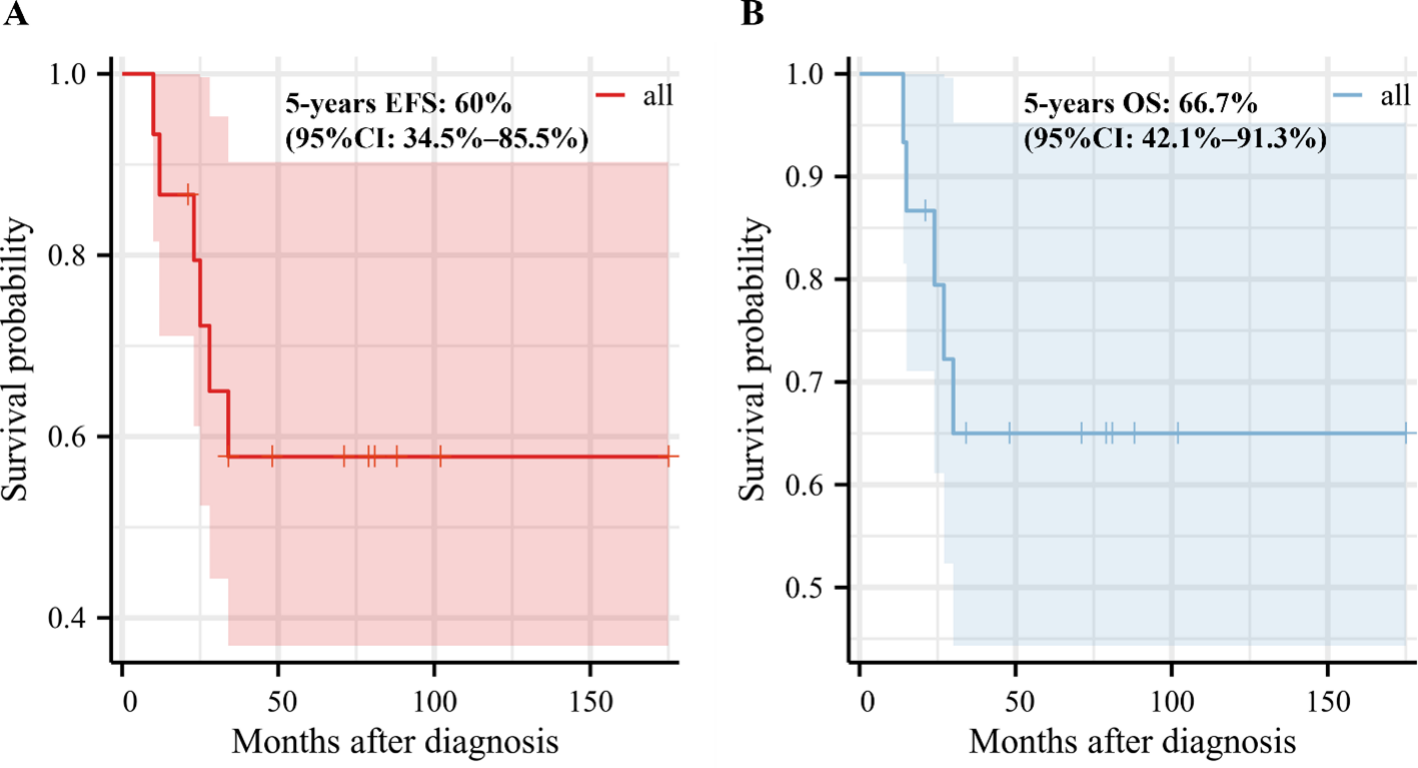
Kaplan–Meier survival curves for the entire cohort. **(A)** Five-year EFS was 60.0%. **(B)** Five-year overall survival (OS) was 66.7%.

With a median follow-up of 48 months, the 5-year EFS was 60% (95% CI: 34.5%-85.5%), and the 5-year OS was 66.7% (95% CI: 42.1%-91.3%) (**Figure 1**). The overall relapse/progression rate was 40% (6/15). In the analysis of risk factors, the relapse/progression rate was significantly higher in patients with metastasis at diagnosis compared to those with localized disease (85.7% vs 0%, *P < 0.001*). Similarly, patients with R2 resection had a significantly higher relapse/progression rate than those with R1 and R0 resection (100% vs 10%, *P < 0.001*). Regarding risk stratification, the relapse/progression rate was significantly higher in the high-risk group compared to the intermediate-risk group (75.0% vs 0%, *P = 0.010*). Additionally, patients with regional lymph node involvement showed a higher relapse/progression rate than those without (66.7% vs 22.2%), although the difference was not statistically significant (*P* = *0.123*). For histological subtypes, the relapse/progression rate was 44.4% (4/9) in patients with embryonal RMS and 33.3% (2/6) in those with alveolar RMS. However, this difference was not statistically significant (*P = 1.000*) (**Table 2**).

**Table 2:** Factors associated with relapse/progression.

Variable	n (%)	Relapse/progression (%)	95% CI	P
Total	15	6 (40.0)	34.5~85.5	
Sex				1.000
Male	8 (53.3)	3 (37.5)	10.9~69.2	
Female	7 (46.7)	3 (42.9)	11.6~79.0	
Maximum tumor size				1.000
≤5cm	6 (40.0)	2 (33.3)	6.0~70.0	
>5cm	9 (60.0)	4 (44.4)	16.8~75.6	
Histology				1.000
ERMS	9 (60.0)	4 (44.4)	16.8~75.6	
ARMS	6 (40.0)	2 (33.3)	6.0~70.0	
T status				0.307
T1	6 (40.0)	1 (16.7)	0.4~64.1	
T2	9 (60.0)	5 (55.6)	23.7~83.5	
Regional lymph node involvement				0.123
N0	9 (60.0)	2 (22.2)	4.0~54.0	
N1	6 (40.0)	4 (66.7)	24.1~94.0	
Metastatic status				<0.001
M0	8 (53.3)	0	0~37.5	
M1	7 (46.7)	6 (85.7)	54.3~99.9	
Primary tumor resection				<0.001
R0+R1	10 (66.7)	1 (10.0)	0.2~44.7	
R2	5 (33.3)	5 (100.0)	47.8~100	
Risk group				0.010
Intermediate-risk group	7 (46.7)	0	0~41.0	
high-risk group	8 (53.3)	6 (75.0)	39.0~94.0	
Oberlin score				0.485
≤1 Risk Factor	2 (25.0)	1 (50.0)	1.3~98.7	
≥2 Risk Factors	6 (75.0)	5 (83.3)	36.5~99.2	

## 4 Discussion

In our center, a total of 81 pediatric patients were diagnosed with rhabdomyosarcoma, of whom 15 presented with tumors originating in the trunk and extremities, accounting for 18.5% of all RMS cases. The lower extremity as the predominant site (60%), consistent with published literature (4). The median age at diagnosis was lower than that reported by COG. ERMS was the predominant subtype (4). Although this distribution was inconsistent with previous reports (14), the proportion of alveolar histology in our study was higher than the 14.5% observed in all RMS patients at our center during the same period. Unfortunately, due to limited testing capabilities in the early period, *FOXO1* fusion gene analysis was performed in only a subset of patients with alveolar RMS (4).

The overall prognosis for pediatric trunk and extremity RMS remains unfavorable. In our center, the 5-year EFS and OS are lower than those of the concurrent overall cohort (71.1% and 72.4%) and corroborate previous domestic and international reports regarding the poor prognosis associated with extremity RMS (4). The presence of metastasis at diagnosis was identified as the most critical factor associated with relapse/progression. The metastatic group exhibited a significantly higher relapse/progression rate (85.7%) compared to the localized group. While patients in the intermediate-risk group demonstrated excellent outcomes, the prognosis for the high-risk group remained unsatisfactory. Notably, the Oberlin scoring system showed a trend toward predictive value within the high-risk cohort; patients with a score ≥2 had a relapse/progression rate of 83.3%, a finding consistent with Oberlin et al. (15, 16) initial reports and subsequent international studies.

The completeness of surgical resection is a key determinant of prognosis for localized disease. Our data revealed that patients with R2 resection had a relapse/progression rate of 100%, significantly higher than the 10% observed in the R0/R1 resection group. This underscores the imperative of achieving complete tumor resection, provided that limb preservation is feasible (9). However, given that trunk and extremity RMS often involves critical neurovascular structures, primary radical resection poses significant challenges (9). In this study, 46.7% of patients underwent DPE following neoadjuvant chemotherapy to facilitate tumor shrinkage. This strategy not only improved the rate of R0/R1 resection but also successfully balanced oncological control with limb function preservation (10).

Regional lymph node involvement is a recognized adverse prognostic factor in extremity RMS. In our cohort, the relapse/progression rate for the node-positive (N1) group was 66.7%, compared to 22.2% in the node-negative group. Although this difference did not reach statistical significance, likely due to the limited sample size, the clinical trend is evident. In practice, rigorous evaluation of regional lymph nodes is highly recommended for extremity RMS, particularly in cases of alveolar histology or large tumor volume, to prevent local recurrence resulting from missed diagnoses (17).

In our cohort, while alveolar histology is generally considered a poor prognostic factor, we did not observe a statistically significant difference in relapse/progression rates between ERMS and ARMS (18). This lack of significance is likely attributable to the limited sample size of our study, which may have reduced the statistical power to detect such a difference.

Existing literature consistently identifies metastasis and lymph node involvement as the strongest predictors of poor prognosis in extremity rhabdomyosarcoma. A review of prognostic factors from recent literature is summarized in **Table 3**. Metastasis, in particular, is the most decisive indicator of worst survival, while node-positive status also portends a dismal prognosis (4, 19, 20). Furthermore, surgical margin status (R0 resection) and specific molecular subtypes (*PAX7:FOXO1*) are also critical variables influencing outcomes (14, 21). Our study findings align with these conclusions, and the lack of statistical significance observed in histological subtypes can be attributed to the limited sample size.

**Table 3:** Literature review of prognostic factors in extremity and trunk rhabdomyosarcoma.

Study (Year)	Main conclusion
Jeremiasse et al. (2021) (19)	SLN status (SNP): The strongest prognostic indicator; N1 status associated with extremely poor survival.
Terwisscha van Scheltinga et al. (2020) (20)	Nodal status (N1): 5-year OS 50.4% vs. 80.3% in N0.
de Traux de Wardin et al.(2023) (14)	Fusion type: PAX7:FOXO1 (86% OS) significantly better than PAX3:FOXO1 (36% OS).
Nitzan e Luques A et al. (2015) (21)	90% of patients achieved R0 resection, which is associated with freedom from local recurrence.
OBEROI et al. (2025) (4)	Localized disease: 5-year event-free survival (EFS) 62.6%, 5-year overall survival (OS) 78.7%. Metastatic disease: 5-year EFS 7.7%, 5-year OS 22.7%. Among metastatic disease: lower Oberlin score was associated with better EFS and OS

SNP, sentinel Node Procedure.

This study has several limitations, a small single-center sample size, missing *FOXO1* data in early cases, and treatment heterogeneity over a long study period. Future multi-center collaborative studies with larger samples are needed to establish reliable risk prediction models.

## 5 Conclusion

In summary, pediatric rhabdomyosarcoma of the trunk and extremities is associated with a poor prognosis. Metastasis at initial diagnosis, macroscopic residual disease after surgery, and regional lymph node involvement confer a high risk of relapse or progression. The Oberlin score demonstrated prognostic predictive value for high-risk patients. However, these findings require further validation in studies with larger sample sizes.

## Data Availability

The raw data supporting the conclusions of this article will be made available by the authors, without undue reservation.
