# Supplementary material for: Factors associated with relapse/progression in pediatric trunk and extremity rhabdomyosarcoma
Source: Front Oncol. 2026 Jul 2;16:1866742. doi: 10.3389/fonc.2026.1866742 (PMC13372660; doi:10.3389/fonc.2026.1866742)
Supplement: Supplementary file 1 [file Table1.docx]

Supplementary Material

# Appendix A

Treatment Schema and Chemotherapy Doses.

| Week | Treatment | | | | Chemotherapy Doses |
| --- | --- | --- | --- | --- | --- |
|  | Rs-99 | Rs-2018 | | |  |
|  |  | LR | IR | HR |  |
| 0 |  |  |  |  |  |
| 1 | DV*CP | VAC* | DVAC | D*VAC | V*: vincristine 1.5 mg/m2/d1,8 |
| 2 |  |  |  |  | V: vincristine 1.5mg/m2/d1,8,15(max dose 2mg) |
| 3 |  |  |  |  | D: adriamycin 30 mg/m2/d2,9 |
| 4 | IV*E | VAC* | IVE | I*VE | D*: adriamycin 35 mg/m2/d2,9 |
| 5 |  |  |  |  | C: cyclophosphamide 300 mg/m2/d✕3 |
| 6 |  |  |  |  | C*: cyclophosphamide 1.200 mg/m2/d1 |
| 7 | DV*CP | VAC* | DVAC | V Irin | P: cisplatin 90 mg/m2/d1 |
| 8 |  |  |  |  | I: ifosfamide 1.5 g/m2/d✕5 |
| 9 | EVAL | EVAL | EVAL | EVAL | I*: ifosfamide 1.8 g/m2/d✕5 |
| 10 | IV*E | VAC* | IVE | D*VAC | E: etoposide 100 mg/m2/d✕5 |
| 11 |  |  |  |  | E*: etoposide 100 mg/m2/d✕3 |
| 12 |  |  |  |  | A*: dactinomycin 0.012 mg/kg/d✕5 |
| 13 | DV*CP | VA | DVAC | I*VE | A: 0.045mg/kg/d1 (max dose 2.5mg) |
| 14 |  |  |  |  | Irin: 50mg/m2/d✕5 (max dose 100mg/d) |
| 15 |  |  |  |  | Sirolimus: 0.1 mg/kg (Rs-2018 HR group) |
| 16 | IV*E | VA | IVE | V Irin |  |
| 17 | EVAL | EVAL | EVAL | EVAL |  |
| 18 | RT | RT | RT | RT |  |
| 19 | V*CP | VA | VAC* | VAC* |  |
| 20 |  |  |  |  |  |
| 21 |  |  |  |  |  |
| 22 | A*E*V* | VA | AE*V | AEV |  |
| 23 |  |  |  |  |  |
| 24 |  |  |  |  |  |
| 25 | V*CP | VA | VAC* | VAC* |  |
| 26 |  |  |  |  |  |
| 27 | EVAL | EVAL | EVAL | EVAL |  |
| 28 | A*E*V* |  | AE*V | AEV |  |
| 29 |  |  |  |  |  |
| 30 |  |  |  |  |  |
| 31 | V*CP |  | VAC* | VAC* |  |
| 32 |  |  |  |  |  |
| 33 |  |  |  |  |  |
| 34 | A*E*V* |  | AE*V | AEV |  |
| 35 |  |  |  |  |  |
| 36 | EVAL |  | EVAL | EVAL |  |
| 37 | V*CP |  |  |  |  |
| 38 |  |  |  |  |  |
| 39 |  |  |  |  |  |
| 40 | A*E*V* |  |  |  |  |
| 41 |  |  |  |  |  |
| 42 |  |  |  |  |  |
| 43 | V*CP |  |  |  |  |
| 44 |  |  |  |  |  |
| 45 |  |  |  |  |  |
| 46 | A*E*V* |  |  |  |  |
| 47 |  |  |  |  |  |
| 48 |  |  |  |  |  |
| 49 | V*CP |  |  |  |  |
| 50 |  |  |  |  |  |
| 51 |  |  |  |  |  |
| 52 | A*E*V* |  |  |  |  |
| 53 |  |  |  |  |  |
| 54 | EVAL |  |  |  |  |
